# A phase III multisite randomised controlled trial to compare the efficacy of cannabidiol to placebo in the treatment of cannabis use disorder: the CBD-CUD study protocol

**DOI:** 10.1186/s12888-024-05616-3

**Published:** 2024-03-04

**Authors:** Anjali K. Bhardwaj, Llew Mills, Michael Doyle, Arshman Sahid, Mark Montebello, Lauren Monds, Shalini Arunogiri, Paul Haber, Valentina Lorenzetti, Dan I. Lubman, Peter Malouf, Mary E. Harrod, Adrian Dunlop, Tom Freeman, Nicholas Lintzeris

**Affiliations:** 1 Faculty of Medicine, University of Sydney, Camperdown, NSW Australia; 2 Drug and Alcohol Services, South East Sydney Local Health District, Sydney, NSW Australia; 3 Drug and Alcohol Services, North Sydney Local Health District, St Leonards, NSW Australia; 4 Centre for Addiction and Mental Health, Turning Point, Victoria, Australia; 5 Drug Health Services, Sydney Local Health District, Sydney, Australia; 6 Faculty of Health Science, Australian Catholic University, Victoria, Australia; 7 Medicine and Health, University of New South Wales, Sydney, NSW Australia; 8 NSW Users and AIDS Association, Sydney, NSW Australia; 9 Drug and Alcohol Clinical Services, Hunter New England Local Health District, Newcastle, NSW Australia; 10 Addiction and Mental Health Group, University of Bath, Bath, UK

**Keywords:** Cannabis, Cannabis withdrawal, Cannabis use disorder, Cannabidiol, Marijuana, Study protocol

## Abstract

**Background:**

Cannabis use disorder (CUD) is increasingly common and contributes to a range of health and social problems. Cannabidiol (CBD) is a non-intoxicating cannabinoid recognised for its anticonvulsant, anxiolytic and antipsychotic effects with no habit-forming qualities. Results from a Phase IIa randomised clinical trial suggest that treatment with CBD for four weeks reduced non-prescribed cannabis use in people with CUD. This study examines the efficacy, safety and quality of life of longer-term CBD treatment for patients with moderate-to-severe CUD.

**Methods/Design:**

A phase III multi-site, randomised, double-blinded, placebo controlled parallel design of a 12-week course of CBD to placebo, with follow-up at 24 weeks after enrolment.

Two hundred and fifty adults with moderate-to-severe CUD (target 20% Aboriginal), with no significant medical, psychiatric or other substance use disorders from seven drug and alcohol clinics across NSW and VIC, Australia will be enrolled.

Participants will be administered a daily dose of either 4 mL (100 mg/mL) of CBD or a placebo dispensed every 3-weeks. All participants will receive four-sessions of Cognitive Behavioural Therapy (CBT) based counselling. Primary endpoints are self-reported cannabis use days and analysis of cannabis metabolites in urine. Secondary endpoints include severity of CUD, withdrawal severity, cravings, quantity of use, motivation to stop and abstinence, medication safety, quality of life, physical/mental health, cognitive functioning, and patient treatment satisfaction. Qualitative research interviews will be conducted with Aboriginal participants to explore their perspectives on treatment.

**Discussion:**

Current psychosocial and behavioural treatments for CUD indicate that over 80% of patients relapse within 1–6 months of treatment. Pharmacological treatments are highly effective with other substance use disorders but there are no approved pharmacological treatments for CUD. CBD is a promising candidate for CUD treatment due to its potential efficacy for this indication and excellent safety profile. The anxiolytic, antipsychotic and neuroprotective effects of CBD may have added benefits by reducing many of the mental health and cognitive impairments reported in people with regular cannabis use.

**Trial registration:**

Australian and New Zealand Clinical Trial Registry: ACTRN12623000526673 (Registered 19 May 2023).

## Introduction

### Cannabis use disorder

Cannabis is the third most widely used drug in the world, after tobacco and alcohol, with an estimated 209 million persons, or 4.1% of the global adult population having used cannabis in the previous year (2020), Cannabis use has increased by 23 per cent between 2010 and 2020 [1]. Worryingly, between 9 and 22% of people who use cannabis will develop moderate or severe cannabis use disorder (CUD) [2] signifying ongoing cannabis use despite clinically significant impairment in health and social function [2]. The most recent global estimate suggests approximately 22.1 million persons met diagnostic criteria for CUD in 2016 (289.7 cases per 100,000 people) [3, 4].

CUD is associated with an increased risk of numerous psychosocial outcomes, including: (i) mental health problems (e.g., anxiety, depression, psychosis, suicide); (ii) physical illness (e.g. respiratory, cardiovascular disease, cancer); (iii) cognitive impairment (e.g., verbal learning, memory and attention); (iv) impaired brain development with prenatal or adolescent exposure; (v) social harms (e.g. crime, employment, parenting, financial impacts); and (vi) motor vehicle accidents [5].

### Treatment for CUD

Existing treatments for CUD have modest outcomes. Reviews of psychosocial interventions (e.g. cognitive behavioural therapy (CBT), motivational enhancement therapy) [6] and acute withdrawal management [7] indicate that over 80% of patients relapse within 1–6 months of attempting treatment [8–11]. In substance use disorders other than CUD, treatment outcomes are generally optimised when combining medications with psychosocial interventions [12]. Despite examining a wide variety of medications, there are no registered pharmacotherapies for treating CUD [13–15].

There is increasing interest in the use of cannabinoid medications to treat CUD. Promising results have emerged in RCTs with delta-9-tetrahydrocannabinol (THC)-based medications (e.g., nabiximols [16], a 1:1 ratio of THC and CBD) and synthetic THC-based medications (e.g., dronabinol, nabilone) [16, 17]. However, many individuals may not be attracted to cannabinoid ‘agonist’ therapy with THC-based medications as they may have intoxicating, psychotogenic, anxiogenic and addictive properties. Thus, there is growing interest in the potential of non-intoxicating cannabinoids, such as CBD, in the treatment of CUD.

### Cannabidiol and CUD

Cannabidiol (CBD) is one of the many cannabinoids found in the *Cannabis sativa* plant. It has diverse and multiple molecular targets [18] and anti-inflammatory, anxiolytic, anticonvulsant and antipsychotic properties [18–24]. Importantly in the context of CUD, CBD is a negative allosteric modulator of the activity of cannabinoid type 1 receptors within the central nervous system, restricting the ability of THC to bind to these receptors, thus reducing THC action [25]. CBD does not cause intoxication, dependence, or withdrawal on discontinuation, and does not result in positive results in urine or saliva tests used to detect cannabis use [26, 27]. Meta-analyses of clinical trials indicate CBD has a good safety profile [28], including in cannabis-using populations [29], and low oral bioavailability (approximately 6%) with a half-life of 18–32 h that permits once daily dosing [30].

CBD has shown promise in animal studies modelling addiction to a range of other substances, with reductions in self-administration of alcohol, opioids, cocaine, and methamphetamine [31]. Endocannabinoids are important regulators of the brain pathways that mediate neurodevelopment, drug-reward and addiction[32, 33]. In human studies examining other addictive drugs, CBD has been found to significantly reduce cue-induced craving and anxiety during abstinence from heroin [34] and cigarette use among tobacco smokers [35], but was not effective in reducing relapse or cravings in a placebo-controlled randomised trial for cocaine dependence [36].

There has been considerable scientific discussion in recent years about the promise of CBD as a treatment for CUD [37, 38], summarised in a recent review: “According to the previous evidence, it seems that CBD could play a crucial role in the management of CUD [38]”. Preclinical studies suggest that CBD administered to cannabis-dependent rodents reduces the severity of spontaneous withdrawal from THC [39–41]. Despite this, there has been little rigorous clinical research to date examining CBD as a treatment for CUD in humans. Early open-label case studies involving 10 participants with severe CUD indicate that CBD may ameliorate cannabis withdrawal severity and improve anxiety and sleep [39, 42, 43]. These studies used doses varying from 18 to 1200 mg daily but had methodological limitations that limit conclusions regarding efficacy.

A recent Phase 2a adaptive Bayesian RCT [44] demonstrated the promise of CBD for moderate-to-severe CUD and determined suitable doses for further investigations. 82 outpatients diagnosed with moderate-to-severe CUD were randomised to four-weeks of oral placebo (*n* = 23), 200 mg CBD (*n* = 12), 400 mg CBD (*n* = 24), or 800 mg CBD (*n* = 23), each receiving six sessions of motivational interviewing The 200 mg dose arm was eliminated as it was not efficacious following interim analyses. Both 400 mg and 800 mg groups were more efficacious than placebo in reducing cannabis use indicated by self-reported cannabis-free days and urinary carboxy-THC (THC-COOH), the inactive metabolite of THC excreted in urine. Doses were well tolerated with no serious adverse events. Reductions in cannabis use persisted 20-weeks after the four-week intervention in the 400 mg, but not the 800 mg group suggesting that further exploration of the 400 mg dose is warranted. However, the study was not powered to demonstrate efficacy, so larger RCTs are also necessary. The relatively high rates of relapse to heavy cannabis use at follow-up may be attributed to the brief treatment duration (4-weeks) examined in the phase 2a RCT. Indeed, in our previous 12-week RCT of nabiximols, we demonstrated that the full extent of reductions in cannabis use were not achieved until at least week 8 [16]. This suggests a prolonged duration, such as, 12-weeks of CBD and counselling may achieve better outcomes.

In their recent 12-week exploratory, observational, non-randomised, open-label study, Cleirec and colleagues [45] investigated the therapeutic potential of inhaled CBD amongst 20 patients, administered through an electronic vaping device, for the treatment of moderate-to-severe CUD. The average daily dose of inhaled CBD was 216 mg (equivalent to approximately 600–700 mg oral CBD). With a flexible dosing regimen and no formal counselling, the study demonstrated promising outcomes, including a notable 30% (*n* = 6) of participants achieving a 50% reduction in days of cannabis use, and 15% (*n* = 3) reporting complete abstinence by the end of the intervention. The absence of significant adverse events or the need for rescue medications further supports rigorous clinical trials examining the efficacy of CBD for CUD.

Fortin and colleagues [46] recently reported findings from an online anonymous survey of French residents who reported having used CBD within the past month. Respondents reported using CBD primarily to reduce their use of (illicit) cannabis. Of these, 59% (61/105) reported that their CBD use led to a large reduction in illegal cannabis consumption, 35% a moderate reduction, 6% no reduction, and 1% a moderate increase. While the study is limited by the self-report nature of the data and the inherent sampling biases of online surveys, the study provides a consumer perspective of the promise of CBD for treating CUD, complementing findings from the aforementioned preclinical and clinical studies.

In addition to CBD’s potential to facilitate a reduction in cannabis use, prolonged high-dose CBD usage in individuals with CUD may offer an additional advantage—potentially mitigating the adverse cognitive and mental health effects of long-term THC exposure [40, 41]. Animal studies indicate CBD reverses THC-induced memory deficits, conditioned place aversion and decreased social interaction [26]. In a human laboratory study, pre-treatment with CBD reduced acute THC-induced persecutory symptoms and hippocampal-dependent memory impairment [40]. In an open-label study, 10-weeks of daily oral CBD (200 mg) was associated with reduced cognitive deficits, psychotic-like and depressive symptoms and increased hippocampal volumes in chronic cannabis users (daily or near-daily use) (despite continued cannabis use), with the greatest benefits seen in those with cannabis dependence [47]. In the same trial, hippocampal and amygdala functional connectivity with other cortical regions (precentral and lingual gyrus, respectively), changed from pre-to-post intervention, with strong effect sizes (*d* > 1) [48]. However, in a Phase 2a RCT, CBD was not found to significantly impact cognition relative to placebo, except in the 800 mg group [49], although the study was underpowered. Nevertheless, the above studies suggest that the anxiolytic, antipsychotic and neuroprotective effects of CBD may improve the psychological, cognitive and brain health commonly related to long-term cannabis use [24]. This may be an added benefit of using high-dose CBD in people seeking treatment for CUD and identifies potential ‘secondary outcomes’ for future studies.

### What are suitable primary endpoints for clinical trials of CUD treatment?

A challenge in embarking on clinical trials for substance use disorder is choosing a primary endpoint. Whilst historically abstinence (cessation of all use) has often been used in cannabis treatment research, there is increasing recognition that abstinence may not be the primary goal of treatment for patients who use cannabis. A recent consensus expert panel identified suitable outcomes when undertaking clinical trials for the treatment of CUD [50], recommending primary outcomes of (a) self-reported frequency of use, using the Time Line Follow Back (TLFB) method [51]; (b) biological assessment of cannabis use, with urinalysis of the metabolite THC-COOH or oral fluid detection of THC; and (c) severity of CUD, using a structured instrument measuring DSM-5 Criteria (e.g. Mini-International Neuropsychiatric Interview (MINI) [52]). In line with these recommendations, and consistent with the previous Phase 2a RCT, we propose to use two primary endpoints to measure illicit cannabis use: (1) self-reported ‘cannabis-free days’ and (2) urinary THC-COOH, across the 12-week treatment period of the study, alongside a range of secondary outcome measures.

### Cannabis use in Indigenous Australian populations

Cannabis use is more prevalent among Aboriginal and Torres Strait Island people than non-Indigenous Australians. Data from the 2019 National Aboriginal and Torres Strait Islander Health Survey [53] indicated 24% (31% males, 18% females) of Indigenous people aged ≥ 15 years reported cannabis use in the past year, an increase from 19% in the corresponding 2012–2013 survey, and 30% higher than non-Indigenous Australians [53]. The elevated prevalence of illicit drug use among Indigenous Australians could be attributed to personal and familial factors, including intergenerational trauma from colonisation and experiences of racism. Societal-level influences such as persistent social and economic marginalisation contribute significantly to the increased likelihood of substance use amongst Indigenous Australians [1, 2].

Not only are prevalence rates of CUD higher, but the harms also resulting from cannabis use are greater in Indigenous Australian communities. Indigenous Australians are five times more likely to be hospitalised for CUD than non-Indigenous Australians [54] and six times more likely to seek treatment for cannabis use than non-Indigenous Australians when adjusted for age [2].

Yet despite the high prevalence of cannabis use and related harms in Indigenous Australians, to date, there have been no clinical treatment trials for CUD among Indigenous Australian populations. This study aims to ensure that a representative proportion of Indigenous Australian participants, with a target of 20% of the total sample, are recruited to the study. The target reflects the proportion of Indigenous Australian clients attending for cannabis treatment in participating study sites, with representation of Aboriginal researchers, health workers and consumers at all levels of the project governance (see Methods).

### Summary

Cannabis Use Disorder poses significant health and social risks. Existing psychosocial treatments for CUD have modest effects, prompting the exploration of effective medications. CBD has emerged as a promising treatment for CUD, backed by preclinical evidence, and pilot data from a recent Phase 2a RCT. CBD offers additional advantages by potentially alleviating the mental health, and cognitive impairments associated with prolonged cannabis use.

## Methods

### Research hypothesis and study aims

The *research hypothesis* is that CBD, compared to placebo, will achieve statistically and clinically significant reductions in cannabis use, as measured by the number of self-reported cannabis-free days and urinary THC-COOH levels, among treatment-seeking patients with moderate-severe CUD.

The *primary aim* of the CBD-CUD study is to examine the efficacy of CBD, compared to placebo, in reducing cannabis use (as measured by self-report and quantitative measures of cannabis metabolites (THC-COOH) in urine drug screens) during treatment (Weeks 1–12) in participants seeking treatment for moderate-severe CUD, when used in combination with psychological interventions.

*Secondary aims* include examination of (i) safety, (ii) other cannabis related measures (e.g., cannabis withdrawal and cravings, cannabis-related problems); (iii) tobacco and other substance use; (iv) health and quality of life (QoL) measures, (v) patient experience measures; (vi) treatment retention rates; (vii) cognitive performance; (viii) post-treatment (Week 24) cannabis use, health outcomes and QoL measures.

### Study design

**Fig. 1:**
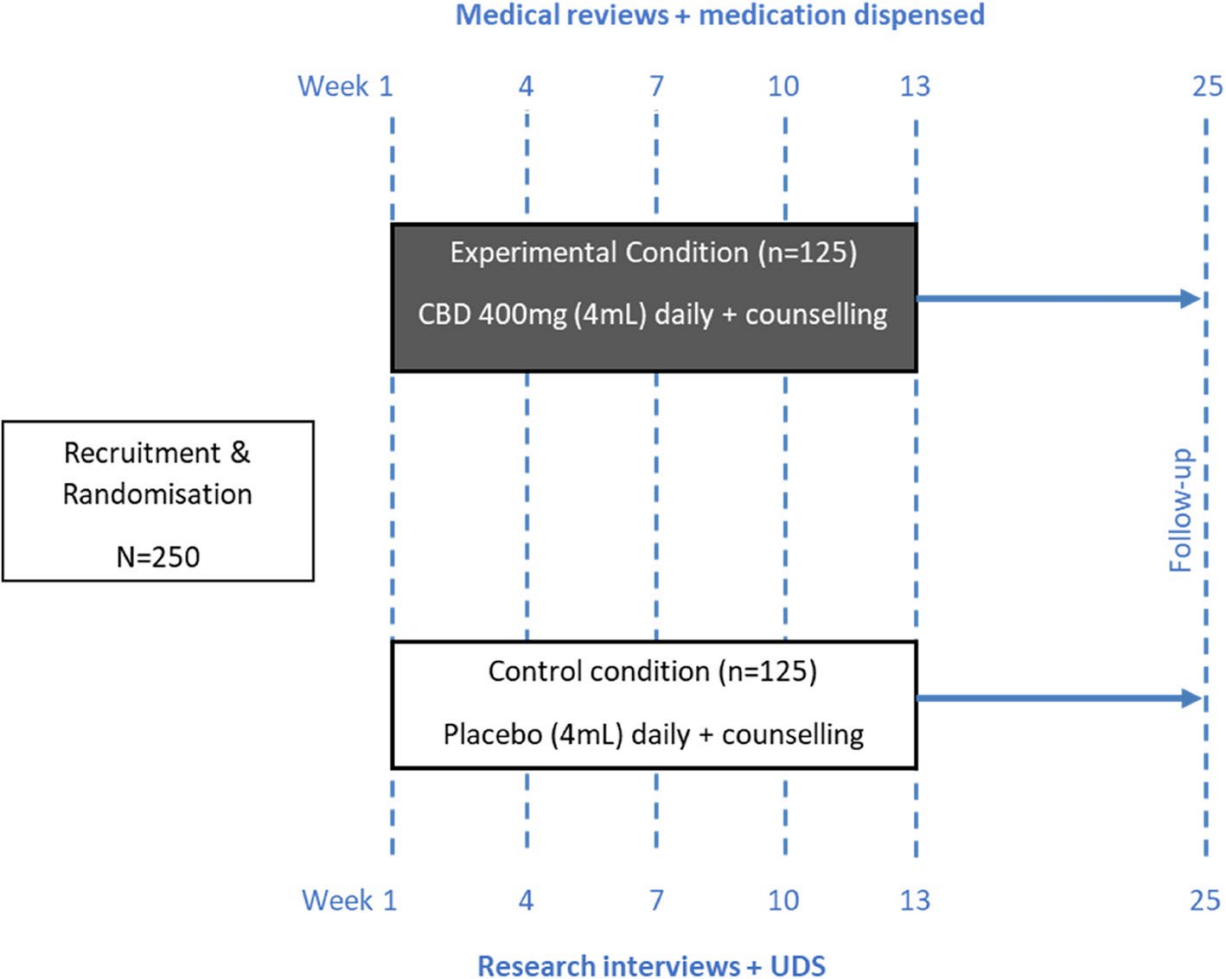
Overview of study design

The study is a parallel group prospective double-blind Phase 3 randomised controlled trial comparing a 12-week treatment period of oral CBD (400 mg daily) (Experimental) to placebo (Control), with both groups receiving 4 sessions of manualised CBT-based counselling. Research interviews will be conducted at baseline (week 1), 3-weekly during the study intervention (weeks 4, 7, 10 and 13) and 12-weeks after the end of treatment (week 25) (Fig. 1). The study will use a modified intention-to-treat analysis. The expected number of participants is 250, of which we estimate approximately 20% (*n* = 50) will be of Indigenous background. The study will also include qualitative interviews with Indigenous Australian participants in both control and intervention groups (a total of *n* = 15–25 Indigenous Australian participants) to examine their experiences in the study.

### Ethical statement

The study will be conducted in accordance with the *National Statement on Ethical Conduct in Human Research* (2007), the *CPMP/ICH Note for Guidance on Good Clinical Practice* and consistent with the principles that have their origin in the Declaration of Helsinki. The study was approved by the Sydney Local Health District Human Research Ethics Committee (no 2022/ETH02467) and the Aboriginal Health and Medical Research Council’s Human Research Ethics Committee (no 2110/23). The project has an Aboriginal Reference Group that oversees all aspects of the study, including data collection and analysis as they relate to Indigenous Australians. The study has been registered on the Australian and New Zealand Clinical Trial Registry (ACTRN12623000526673).

### Setting and study sites

The multicenter trial will be coordinated from the Specialty of Addiction Medicine, Faculty of Medicine and Health, University of Sydney (study sponsor). Treatment will be provided at seven specialist addiction outpatient treatment centers: four in Sydney, one in Newcastle and two in Melbourne, Australia.

### Participants and recruitment

#### Eligibility criteria

The target study population is treatment-seeking adults with moderate to severe CUD under conditions of informed consent. Eligibility will be assessed by an Addiction Medicine or Psychiatry credentialed Study Medical Officer (SMO).

Inclusion criteria*:*

1. Aged 18 to 65 years.
2. Meeting DSM-5 criteria for moderate or severe CUD (≥ 4/11 criteria) [2], with recent frequent cannabis use (≥ 4 days per week in the preceding 4 weeks).
3. Willing and able to provide informed consent to study procedures.
4. Proficient in English at a conversational level sufficient to participate in a counselling intervention.

*Exclusion criteria* aim to exclude individuals with conditions that jeopardise safety or confound data interpretation:

1. Prescribed medicinal cannabis products (e.g., CBD, THC) for any indication in the previous 4 weeks.
2. Another active (past year) moderate-severe substance use disorder other than tobacco; determined on clinical assessment using DSM-5 criteria.
3. Active or severe medical (e.g., pain, epilepsy, cardiovascular disease) or psychiatric (e.g., psychosis, severe affective disorder) conditions based on clinical assessment.
4. Moderate to severe hepatic disease (transaminase elevations > 3 times, bilirubin > 2 times upper normal limits at screening).
5. Pregnant or lactating women (based on urine β-hCG at screening).
6. Hypersensitivity to CBD or any excipients of Investigational Product.
7. Using medications with known drug-drug hepatic CYP-450 interactions with CBD: 3A4, (e.g., carbamazepine, fluvoxamine, methadone), 2C19 (e.g., rifampin); CYP2B6 (e.g., bupropion), CYP2C9 (e.g., warfarin).
8. Not available during treatment or follow-up (e.g., travel, impending residential detoxification or residential rehabilitation admission, impending imprisonment).
9. Court-mandated treatment requiring abstinence from drugs.
10. Current active (counselling and/or medication-based) treatment for CUD.
11. Received an investigational medicinal product within the last 4 weeks (or 5 half-lives if using long-acting investigational drugs).

#### Participant numbers

Sample size calculations are based on the analysis of the primary outcome, that is, the difference between placebo and CBD groups in total number of Cannabis-free Days over the 12-week intervention period. Ferguson has suggested that the minimum effect size (Cohen’s *d*) for an effect of practical clinical significance is 0.4167 [55]. To achieve 90% power (two-tailed) and α = 0.05, a total of *N* = 250 (*n* = 125 per group) participants are needed to detect a between-group effect size of *d* = 0.41. Of the target 250 sample, it is estimated approximately 20% (*n* = 50) of the study sample (*N* = 250) will be Indigenous Australians.

Participants who discontinue study procedures after commencing study interventions (medication dispensed on Day 1) will not be replaced in the study but will be included in the modified intention-to-treat analyses. Participants enrolled and randomised, but do not commence any treatment (no medication dispensed or other clinical interventions) will not be included in the final analysis.

#### Recruitment, screening and assessment

Participants will be recruited from people seeking treatment at participating study sites, and/or people interested in the study in response to study advertisements at local health services, social media, and clinical trial recruitment platforms. On initial contact with the service, potential participants will be informed of the study and if interested, referred to a site coordinator to complete telephone screening. Following telephone screening, potentially eligible participants will be scheduled a face-to face assessment with a Study Medical Officer (SMO) to confirm eligibility. Potential participants will sign a medical screen consent form prior to the SMO completing a structured history, clinical examination, and any laboratory investigations with the participants. The SMO will also explain the study requirements to the potential participant and explain the study medication and any potential side effects. Eligible participants are scheduled an appointment (Week 1, Day 1) to attend for enrolment into the study. For those participants who are not eligible or choose not to participate in the study, alternative treatment options will be organised in collaboration with the patient, as clinically appropriate.

#### Informed consent, randomisation and blinding

Written informed consent is obtained on Week 1, Day 1 of the study prior to the commencement of all subsequent study procedures. Consent is obtained with the site coordinator independent of treating clinicians, to minimise ‘pressure’ to participate in the study.

The randomisation schedule has been developed by an independent statistician, with eligible participants randomised in a 1:1 ratio between groups using variable block randomisation to help maintain blinding, with subjects stratified by (a) site (to achieve approximately equal numbers of active and placebo at each site) and (b) Indigenous Australian status (to allow direct between-group statistical comparisons within Indigenous Australian participants).

Participants, clinicians, and researchers involved in service delivery, data collection and analysis will remain blinded to study conditions using matched placebos manufactured by the same manufacturer. CBD and placebo will be packaged in identically labelled containers with the participant’s ID number and site. Aside from site trial pharmacists (who have no direct contact with participants), all other members of the clinical or research teams will be blinded to group allocation.

Unblinding will occur after all data are collected, entered, cleaned and the trial database has been locked. In circumstances where allocation needs to be unblinded (e.g. severe adverse event), the principal investigator will authorise the local site investigator to break the blind (via the site trial pharmacist).

### Interventions

#### Medications

The experimental condition will receive 12 weeks of CBD oral 400 mg daily, administered as 200 mg liquid administered twice a day (BD). The CBD used in the trial is a plant-extracted pharmaceutical product (registered in Australia as Epidyolex® for the treatment of paediatric epilepsy), and is an oral liquid (clear, colourless to yellow solution) containing 100 mg per ml, dispensed in 105 ml bottles. The placebo is identical in composition and appearance (with the exception of the CBD). Both CBD and placebo are manufactured and supplied by Jazz Pharmaceuticals.

The dose is selected based on the findings of the Phase IIa RCT [29] that identified a daily dose of 400 mg CBD being more efficacious than placebo at reducing cannabis use during 4-week treatment and follow up.

Nicotine dependent participants will be offered smoking cessation counselling during the trial, with prescriptions and supply of nicotine replacement therapy (NRT) either in the form of 16-h topical patches (7, 14 or 21 mg) and/or nicotine chewing gum or lozenges provided.

#### Counselling

All participants will receive four structured 40–50-min counselling sessions over the 12-week medication phase, based on cognitive behavioural therapy (CBT) and motivation enhancement for relapse prevention, consistent with identified ‘best practice’ for cannabis cessation interventions [56]. Available evidence suggests 4-sessions of CBT deliver comparable outcomes to 6 or more sessions for treating CUD [57]. Counselling will be delivered by psychologists experienced in CUD treatment and trained to deliver manualised counselling interventions. Study Counsellors will keep a log of attendance at counselling sessions.

#### Clinical reviews

Participants will have 3-weekly medical reviews with the SMO over the 12-week intervention (Weeks 1, 4, 7, 10 and Week 13). At each appointment, the SMO will review cannabis and other substance use since the last appointment, other health and social issues, and client goals, complete Concomitant Medications and Adverse Events assessments, collect UDS, and supply medications dispensed by the trial pharmacist.

### Outcome measures

The *primary outcomes* are illicit cannabis use during the 12-week intervention period, operationalised using two endpoints:

1. *Cannabis-free Days* over the 12-week intervention period, producing a continuous measure between days 1 and 85. Details regarding number of days of cannabis use will be collected at each research interview (baseline week 1, weeks 4, 7, 10, week 13 and 25) using the Time Line Follow Back (TLFB) approach, a reliable and validated measure of cannabis use, particularly when combined with biological assays (e.g. UDS) and confidentially reported to independent researchers^74^.
2. *Urinary quantitative analysis of THC-COOH (creatinine adjusted)*. Urine samples will be collected at weeks 1, 4, 7, 10, 13 and 25, and analysed using liquid chromatography-tandem mass spectrometry (LCMS). As THC-COOH can remain ‘positive’ using qualitative thresholds (e.g. 20 ng/ml in LCMS assays) for more than 30 days after abstinence in chronic heavy cannabis users [58], we will analyse quantitative levels of THC-COOH (creatinine adjusted) to detect differences in cannabis use between the two study groups, replicating the approach used in the pilot RCT [29] (see below).

Secondary outcomes (Table 1) include a range of measures that relate to cannabis use (including rates of abstinence or reduced frequency of cannabis use, cannabis withdrawal and cravings, cannabis related problems, severity of CUD), safety (adverse events), health outcomes (including mental health, physical health, QoL), consumer experience of the medication, cognitive performance, other substance use and post-treatment outcomes (12 weeks after the intervention). The relationship between experiences of racial discrimination (using the modified Everyday Discrimination Scale) and outcomes for Indigenous Australians will also be explored.

**Table 1:** Table of all primary and secondary outcome measures included in study

Domain	Name	Description of measure	Administration timepoint
**Basic**	Demographics	7 Questions: basic demographics including: age, gender, sexuality, Aboriginal and Torres Strait Islander background, education level, employment status, relationship status and living situation. Categorical data, excluding age which is discrete numeric	Research Interview: Week 1 (baseline) Day 1
**Cannabis Use**	Modified Time Line Follow Back (TLFB) [64]	Cannabis free days, self-reported using a modified timeline follow back (TLFB). Week 1 and 25 interviews will ask about cannabis use in the preceding 4-weeks; week 4, 7, 10 and 13 interviews will ask about the preceding 3-weeks. Bounded count; number of days abstinence (out of 84)	Research Interview: Week 1 (baseline), 4, 7, 10, 13 and 25
	Diagnostic & Statistical Manual of Mental Disorders (DSM-V) [65]	Severity of Cannabis Use Disorder in the prior 12-weeks using the modified (DSM-5 criteria) MINI scale. Number of DSM-5 CUD criteria met out of 11 (Mild CUD: 2–3; Moderate CUD: 4–5; Severe CUD: 6 +) Bounded count, number of DSM-5 CUD criteria met (out of 11)	Research Interview/ Clinical review: Week 1 (baseline), 13 and 25
	Cannabis Use History	7 semi-structured questions exploring: history and frequency of cannabis use and any previous attempts at reducing cannabis use. Continuous	Research Interview/ Clinical review: Week 1 (baseline)
	The Substance Use Goals Questionnaire (SUG)	Bespoke scale developed by addiction medicine specialists involved in the CBD-CUD Study. Participants goal towards their cannabis use. Measured using a self-reported substance use goals questionnaire: 2 items with 5 response categories. 5-level ordinal	Research Interview: Week 1 (baseline), 4, 7, 10, 13 and 25
	Rates of Abstinence	The proportion of participants who achieve pre-identified levels of abstinence from cannabis use (weekly rates), and a 50% reduction in cannabis use days compared to the pre-treatment 4-week period. Binary categorical (non-abstinent vs abstinent)	Research Interview: Week 1 (baseline), 4, 7, 10, 13 and 25
	Cannabis Withdrawal Scale (CWS) [51]	19 items on a 10-point Likert Scale, with 0 = Not at all to 10 = Extremely. The CWS is used as a diagnostic instrument to assess severity of cannabis withdrawal symptoms. Self-report over the last 24 h in 8 domains; irritability, depression, anxiety, cannabis cravings, physical symptoms, sleep difficulty, restlessness and appetite. Continuous (average item score as a 0–10 range, converted to a z-score)	Research Interview: Week 1 (baseline), 4, 7, 10, 13 and 25
	Marijuana Craving Questionnaire – short form (MCQ) [66]	12 items on a 7-point Likert scale, with 1 = strongly disagree to 7 = strongly agree. The MCQ is a self-report instrument that assesses marijuana craving along 4 dimensions; compulsivity, emotionality, expectancy and purposefulness. Continuous (average item score as a 0–10 range, converted to a z-score)	Research Interview: Week 1 (baseline), 4, 7, 10, 13 and 25
	Marijuana Problems Scale (MPS) [67, 68]	19-item MPS measuring potential negative effects of marijuana on social relationships, self-esteem, motivation and productivity, work and finances, physical health, memory impairment and legal problems on a 3-point Likert scale, with 0 = no problem, 1 = minor problem or 2 = serious problem. Continuous (total count of problems (0–19) or as a summed total of severity rating (0–38)	Research Interview: Week 1 (baseline), 13 and 25
	Urine Drug Screen	Urinary Drug Screen (UDS) collected to analyse for quantitative creatinine adjusted THC-COOH and CBD-COOH. Continuous (ng/ MoL)	Research Interview: Week 1 (baseline), 4, 7, 10, 13 and 25
**Other Substance Use**	Australian Treatment Outcomes Profile (ATOP) [69]	3-Part Questionnaire: 1. Substance use; items refer to substance use over the last 4-weeks, in particular: alcohol, amphetamines, benzodiazepines, heroin, other opioids, cocaine, other substances and tobacco 2. Injecting risk behaviour; 2-items exploring injecting behaviour in the last 4-weeks 3. Health and Well-being; 11-items investigating the participants’ accommodation arrangements, psychological and physical health and quality of life Bounded count (days of use/ abstinence in previous 28-days)	Research Interview: Week 1 (baseline), 4, 7, 10, 13 and 25
	Fagerstom Test for Nicotine Dependence (FTND) [70]	6-item questionnaire that measures nicotine dependency, with a total available score of 10. Continuous (range 0–10, converted to z-score)	Research Interview: Week 1 (baseline), 4, 7, 10, 13 and 25
**Mental and Physical Health**	Psychotomimetic States Inventory (PSIa) [71]	48-item questionnaire with subscales in delusional thinking, perceptual distortions cognitive disorganisation, anhedonia, mania, and paranoia. Continuous (range 0–48, total and subscale scores each converted to z-scores)	Research Interview: Week 1 (baseline), 7, 13, and 25
	PCL-5 for Post Traumatic Stress Disorder (PTSD) [72]	20-item self-report measure of the 20 DSM-5 symptoms of PTSD. The four domains consist of: Re-experiencing, Avoidance, Negative alteration in cognition and mood and Hyper-arousal. Continuous (range 0–80)	Research Interview: Week 1 (baseline), 13 and 25
	Patient Reported Outcomes Measurement Information System (PROMIS-29) v2.1 [58]	29-item self-report measure assessing the past 7-days. The seven domains include: physical function, anxiety, depression, fatigue, sleep disturbance, ability to participate in social roles and activities, pain interference and intensity. A 5-point Likert scale (range 1–5) to measure symptom severity or frequency. Continuous (total and subscale scores converted to a z-scores)	Research Interview: Week 1 (baseline), 4, 7, 10, 13 and 25
**Social/ Other**	Treatment Satisfaction Questionnaire for Medication (TSQM) [73]	14-item questionnaire across four domains focusing on drug effectiveness, side effects, convenience and global satisfaction of the medication. Continuous (range 0–100)	Research Interview: Week 4, 7, 10, 13 and 25
	Drug Effects Questionnaire-5 (DEQ-5) [74]	5-item questionnaire assessing consumer ratings of drug effects. Continuous (range 0–7)	Research Interview: Week 4, 7, 10, 13 and 25
	Testing the Blind	2-questions exploring the participants’ subjective opinion of study drug allocation. Binary categorical (placebo vs CBD) and continuous (% confidence)	Research Interview: Week 1 (baseline), 4, 7, 10, 13 and 25
	Safety Adverse Events	Adverse events assessed with study clinician to assess for any side effects of study drug. Unbounded count	Research Interview: Week 1 (baseline), 4, 7, 10 and 13
	Treatment Retention	Calculated from treatment records and participant withdrawal/ completion form. Discrete numeric (week of dropout)	Clinical review: week 13
	Modified Everyday Discrimination Scale (m-EDS) [75]	10-item instruments based on the Discrimination/ Racism in Everyday Life and 6-item instrument based on Discrimination/ Racism in Healthcare adapted to a diverse Aboriginal and Torres Strait Islander context. Participants are asked to report the extent to which they experience discrimination in different settings, with the response options of ‘not at all’, ‘a little bit, ‘a fair bit’ and ‘a lot’. Continuous	Research Interview: Week 1 (baseline)
	Qualitative research interviews	Qualitative research interviews will be conducted with Indigenous Australian participants who consent to participate. Qualitative methods will collect data on how participants’ experience of racial discrimination, identification with culture and community, and how their social and cultural networks (peers, family and local community) impact on attempts to change their cannabis use, as well as how participants perceive their cannabis use and identify their treatment goals and how participants engage with the study treatment procedures (medication, counselling and the role of Aboriginal Health Workers at study sites	1 interview during weeks 7–13 (flexible)
**Cognitive Assessment**	National Adult Reading Test 17-word version (NART17) [76]	The NART is a prevalent tool used in clinical scenarios to predict an individual’s prior level of intelligence, often crucial in neuropsychological studies and practices. The NART-17 consists of 17 written words in British English which all have irregular spellings. Continuous (scales converted to a z-score)	Research Interview: Week 1 (baseline)
	Rey Auditory Verbal Learning Task (RAVLT) [77]	Test of verbal learning and memory. Retention of a 15-item word list presented on 5 occasions. Number of words recalled on each presentation, following presentation of a novel list of 15 words; and then after brief and long delays recorded. Recognition of target stimuli also assessed. Continuous (scales converted to a z-score)	Research Interview: Week 1 (baseline) and 13
	Eriksen Arrow Flankers (with no-go) [78]	Speed of response to target stimuli flanked with either neutral, congruent or incongruent stimuli (24 each). Random 10% (n = 8) trials require with-holding of response (no-go). Reaction time: errors and false alarms recorded. Continuous (scales converted to a z-score)	Research Interview: Week 1 (baseline) and 13
	N-Back [79]	N-back is a continuous performance task to measure verbal working memory and is dependent on the integration of the frontal and temporal regions, two areas particularly affected by THC. Participants are instructed to monitor a series of stimuli and to respond whenever a stimulus is presented that is the same as the one presented *n* trials previously. Continuous (scales converted to a z-score)	Research Interview: Week 1 (baseline) and 13
	Digit Span Task [80]	Task evaluates working memory. The test consists of two-parts: (1) ‘Digits Forward’, requires the participant to repeat sequences of number in the order given; (2) ‘Digits Backward’, requires participant to recite the numbers in reverse order. Continuous (scales converted to a z-score)	Research Interview: Week 1 (baseline) and 13
	Trail Making Test (TMT) [81]	The TMT is a tool used to gauge an individual’s attention, processing speed and cognitive flexibility. It has two parts, in which the participant is instructed to connect a set of 25 dots as quickly as possible while maintaining accuracy. Continuous (scales converted to a z-score)	Research Interview: Week 1 (baseline) and 13

#### Research interviews

The schedule of trial procedures and assessments for participants, including the timing of research and clinical interventions is shown in Table 2. Participants are scheduled to have interviews with researchers at 3-weekly intervals during the 12-week intervention (Weeks 1, 4, 7, 10 and 13), and again 12 weeks after the intervention (Week 25). These interviews will be face-to-face with a researcher, although they can be undertaken by telehealth if required. All data collected at researcher interviews will be entered directly into an electronic database, REDCap, and kept confidential from treating clinicians. Participants will be reimbursed with shopping vouchers for time, inconvenience, and expenses of attending research interviews [59].

**Table 2:** Schedule of trial procedures and assessments

Assessment/ Procedure	Screening	Intervention Phase					Follow-up
**Week**	**-t**_**1**_	**1**	**4**	**7**	**10**	**13**	**25**
**ENROLMENT**							
Phone screen (eligibility)	X						
Medical screen/assessment (eligibility)^a^	X						
Urine drug screen and βhcG	X						
Blood test (LFTs)	X						
Informed consent		X					
Enrol and Randomisation		X					
**INTERVENTION**							
Medication [CBD or placebo] dispensed		X	X	X	X		
Adverse Events Assessments^b^			X	X	X	X	
Concomitant Medications^b^			X	X	X	X	
CBT counselling intervention^c^			X		X		
**RESEARCH ASSESSMENTS**							
Research Interviews^d^: Collection of UDS, TLFB, Quantity of Cannabis Use, SUG, Rates of Abstinence, PROMIS-29, Testing of the Blind, CWS, MCQ, ATOP, FTND		X	X	X	X	X	X
DSM-5, PTSD, MPQ		X				X	X
TSQM, DEQ-5			X	X	X	X	X
PSIa		X		X		X	X
Treatment Retention						X	
Cognitive assessments^e^: RAVLT, NART-17, Eriksen Arrow Flankers Task, N-Back, Digit Span Task, TMT		X				X	
Interviews with Indigenous Australian participants + m-EDS^f^		X		X			

^a^Medical screening to be conducted within 4 weeks of study enrolment on Week 1, Day 1

^b^Adverse event assessments and concomitant medications are assessed by the study medical officer or clinical nurse specialist

^c^Cognitive behavioural therapy intervention to be conducted by experienced cannabis counsellors. Three sessions to be conducted over the first 6 weeks of treatment and one session between weeks 7–13 of intervention period

^d^All research interviews and cognitive assessments will be conducted by the site coordinator. See Table 1 for descriptions of each research measure

^e^RAVLT: This task assesses verbal learning and memory; NART-17 = National Adult Reading Test: this task assesses pre-morbid IQ; Eriksen Arrow Flankers Flankers: This test assesses both choice reaction time and the ability to ignore distracting but irrelevant information; N-back: this is a working memory task; Digit span task: evaluates working memory; Trail Making Test: assesses attention, processing speed, and cognitive flexibility

^f^Modified Everyday Discrimination Scale (m-EDS) will be conducted at Week 1 baseline and a qualitative research interview with consenting Indigenous Australians will be conducted by the study’s Aboriginal Research Coordinator between weeks 7–13

#### Qualitative interviews with indigenous participants

To gain insights into the experience of Indigenous Australian participants, semi-structured in-depth interviews will be conducted by Aboriginal researchers (part of the study team) at around week seven with Indigenous participants in both control and interventions groups until data saturation occurs—estimated at 15 to 25 participants. These interviews will examine topics such as (a) how participants perceived their cannabis use and identified their treatment goals, and how participants are supported by their family and community; (b) how participants engage with the study treatment procedures (medication and counselling) providing insights into future implementation. The interviews will take approximately 40 to 60 min and be digitally recorded and transcribed. A yarning methodology will be used for the data collection and analysis [60].

### Data management and monitoring

Confidentiality of participant data will be secured by removing all identifiable data and replacing it with a unique identifier. The principal investigator and coordinating researcher will have access to key files that link the unique identifier to identifiable data if unblinding is necessary.

Trial data will be electronically entered and stored on REDCap on the research drive of the University of Sydney, with regular data back-up. After the trial, the data will be stored for a minimum of 15 years in a secured study-specific folder on the research drive of the University of Sydney, and access to de-identified data will be considered upon request by the principal investigator.

An independent Data Safety and Monitoring Committee (IDSMC), comprising of an addiction medicine specialist, a statistician and clinical pharmacologist will oversee the safety monitoring of the trial, involving ongoing reviews of any adverse events arising from the administration of CBD (unblinded data). The committee will also monitor aspects of study integrity and design should any protocol changes need to be made.

### Data analysis

All data analysis will be performed using Bayesian models instead of frequentist. Bayesian methods can quantify evidence for both effects and the absence of effects, are less prone to non-convergence (due to regularisation), and sample from a joint posterior distribution, hence no family- or experiment-wise correction of regression coefficients for multiple comparisons is necessary [61]. We will use a modified intention-to-treat approach for data analysis, with group membership fixed as the medication type (placebo vs CBD) participants receive on their first study day. Missing data will be imputed via hierarchical multiple imputation [62].

#### Primary outcomes

We will model the effects of CBD on number of cannabis free days (out of 84 days) via single-level Gaussian regression with the outcome regressed on the main covariate experimental group (placebo vs CBD). Number of cannabis-free days in the 28 days prior to baseline will be included as a covariate to control for variation in participants’ prior frequency of use entering the study. Two treatment factors that could plausibly influence the primary outcomes and which vary across participants will also be included as covariates: number of counselling sessions attended during the study period (count variable range 0–4), and whether or not NRT was taken (binary variable measured at baseline: did not undertake NRT vs undertook NRT). If residuals are distributed normally, we will report the results from this analysis. If residuals are not distributed normally, we will treat cannabis-free days/84 as a bounded count instead of a numeric variable and use aggregated binomial regression with a logit link.

We will model urinary THC-COOH levels (a continuous outcome, in ng/MoL) via random-slopes mixed-effects models with the group, time (6-level categorical ordered predictor; Weeks 1 (baseline), 4, 7, 10, 13), the group × time interaction, number of counselling sessions attended, and whether or not NRT was undertaken as the fixed factors, and participant ID as the random factor.

#### Secondary outcomes

All repeated measures of secondary outcomes will be modelled using the same approach as for urinary THC-COOH. That is, random slopes mixed-effects models for repeated measures regressions with group, time, the group × time interaction, number of counselling sessions attended, and whether or not NRT was taken as the fixed factors. These models will all be based on the generalised linear model, with link functions differing depending on the form of the outcome, as follows:

- Numeric (e.g., PROMIS-29 scores, marijuana craving questionnaire scores): Gaussian regression with identity link function
- Ordinal (e.g., motivation to change cannabis use): ordinal logistic regression with logit link function
- Bounded count (e.g., severity of CUD) or binary (e.g., participant rating of group allocation): binomial logistic regression with logit link function
- Unbounded count (e.g., adverse event count): negative binomial regression with log link function

See Table 1 for the form of each outcome measure.

Several secondary outcomes are single observations per individual. Group, number of counselling sessions attended, and whether or not NRT was taken will be the sole predictors in these models. Total abstinence from cannabis during weeks 10–13 (non-abstinent vs abstinent) and 50% increase in cannabis-free days during weeks 10–13 relative to cannabis-free days prior to baseline (< 50% reduction vs ≥ 50% reduction) will be modelled with binary logistic regression with logit link function, relative risk of adverse events during the trial period with negative binomial regression with log link function, hazard of treatment dropout via discrete-time hazard model with complementary log–log link function.

#### Statistical methods for Indigenous Australian focussed outcomes

Indigenous Australian and non-Indigenous participants will be compared on baseline participant characteristics (e.g., age, gender, frequency of substance use, scores on quality-of-life scales) via simple regression: Gaussian for continuous measures, Logistic for binary and count variables, and multinomial logistic for multilevel categorical data. For the main study analyses, comparing the frequency of illicit cannabis use between placebo and CBD groups, all participants will be pooled and included in main analyses, irrespective of Indigenous status. However, an additional regression will be performed where Indigenous status and the interaction between Indigenous status and the study drug (Placebo vs CBD), along with the primary predictor study drug, will be included in the regression.

The study will stratify randomisation according to Indigenous status, to achieve an approximately equal number of Indigenous Australian participants on active and placebo conditions, thus requiring no additional statistical procedures beyond those outlined in the previous paragraph.

The effect of the experience of discrimination on outcomes related to cannabis use disorder will be estimated via regressing various outcomes related to cannabis use on scores on the modified Everyday Discrimination Scale (m-EDS). Two regressions will be performed for each outcome, with a different version of the m-EDS as the primary predictor in each: (i) a continuous version of the scale (i.e., total score) and (ii) a three-level categorical version of the scale (no vs low vs moderate-to-high). The outcomes that the m-EDS will be regressed on will be: (i) (baseline characteristics (e.g., years of regular cannabis use, scores on quality-of-life scales), (ii), treatment engagement (e.g., treatment retention, number of counselling sessions) and (iii) outcomes during the trial (e.g., frequency of cannabis use, health measures). As described above, the type of regression will depend on the type of outcome: Gaussian for continuous, logistic for binary or bounded count, ordinal logistic for ordered categorical, and negative binomial for unbounded count. The effect of m-EDS on treatment retention will be estimated via Kaplan Meier plots discrete-time hazard model with complementary log–log link function.

## Qualitative data analysis

The qualitative data collected during week seven of treatment, will be analysed by the Aboriginal investigators and the Aboriginal reference group. The data will initially be deductively coded into a) cannabis use, treatment goals and family and community support and b) experience on the study. Data will then be coded in three stages 1) open coding, 2) axial coding and 3) focused/selective coding [63]. After coding has been completed, the data from the deductive code b) experience on the study will be separated into treatment and control groups. The Aboriginal reference group will then analyse and discuss the data. Any divergences in treatment experiences will be explored. The themes identified will be discussed with the Aboriginal reference group to ensure appropriate interpretation with an Aboriginal lens.

### Study governance

The multisite study will occur across the two most populous states in Australia. It will be coordinated through several governance structures, including an overarching Steering Committee (senior research staff and Study Investigators), a Consumer Advisory Group and an Aboriginal Reference Group.

The Consumer Advisory Group includes a Consumer Researcher (member of project team) and between 8 and 12 people with lived experience of cannabis use and treatment, who advise the project team on the study procedures (including recruitment strategies, treatment, and data collection procedures) and assist in interpretation of findings, and dissemination activities with community groups (e.g., lay summaries of study findings).

The Aboriginal Reference Group includes Aboriginal Investigators, research staff, representatives of Aboriginal Health Workers at participating sites, and representatives of Aboriginal Alcohol and Other Drug (AOD) workers in services not participating in the study (to provide independent community perspectives). The Aboriginal Reference and Consumer Advisory Groups will be consulted to interpret and disseminate study findings.

## Data Availability

No datasets were generated or analysed during the current study.
